# Effects of SiO_2_ and ZnO Nanoparticles on Epoxy Coatings and Its Performance Investigation Using Thermal and Nanoindentation Technique

**DOI:** 10.3390/polym13091490

**Published:** 2021-05-06

**Authors:** Mohammad Asif Alam, Ubair Abdus Samad, Arfat Anis, Manawwer Alam, Mohd Ubaidullah, Saeed M. Al-Zahrani

**Affiliations:** 1 Center of Excellence for Research in Engineering Materials (CEREM), King Saud University, P.O. Box 800, Riyadh 11421, Saudi Arabia; moalam@ksu.edu.sa; 2 SABIC Polymer Research Center (SPRC), Chemical Engineering Department, King Saud University, P.O. Box 800, Riyadh 11421, Saudi Arabia; szahrani@ksu.edu.sa; 3 Department of Chemistry, College of Science, King Saud University, P.O. Box 2455, Riyadh 11451, Saudi Arabia; maalam@ksu.edu.sa (M.A.); mtayyab@ksu.edu.sa (M.U.)

**Keywords:** epoxy, coatings, mechanical, nanoindentation, nanoparticles

## Abstract

Synergistic formulations were developed with nano-pigments, and their effects on the mechanical properties on steel substrates and structures were evaluated. This paper provides a complete analysis of the epoxy coating, focusing on the incorporation of nano-pigments and their synergistic effects in obtaining higher mechanical properties. This study reports the preparation of epoxy nano-silica composites, their characterization, and the development of coatings based on nano-silica and ZnO particles. In this composite, epoxy resin was incorporated with SiO_2_ as the main pigment and ZnO as a synergistic pigment to achieve high-performance epoxy coatings for multiple applications. The mechanical properties of these coatings (ESZ1–ESZ3) were evaluated by nanoindentation, and were used to measure the enhanced durability of nanocomposite coatings developed with synergistic formulations with different types of nanoparticles. Their performance was evaluated before and after exposure to a 3.5% NaCl solution to examine the changes of hardness and elastic modulus. The results showed that the nanoindentation technique, in conjunction with Fourier transform infrared spectroscopy and X-ray diffraction, could examine the durability and predict the service life of nanocomposite coatings. A correlation was observed between the modulus and hardness before and after exposing epoxy composite coatings (ESZ1–ESZ3) to a 3.5% NaCl solution.

## 1. Introduction

The development of high-performance multifunctional coatings with scratch and abrasion resistance and durable coatings for the surface of various steel substrates has remained a challenging task for outdoor and industrial applications. Scratching and abrasion can occur during the pre- or post-delivery handling periods or even during transportation, utilization in agriculture, and the movement of vehicles at rough surfaces. Some special resins, curing agents, and additives are incorporated into the formulations to prevent scratching of metal surfaces. Nowadays industrial and protective coating systems are designed to achieve multi-functions, e.g., protection, high mechanical properties, fire resistance, and corrosion resistance [1,2,3,4]. The protective coating system has significant importance, particularly under service conditions of exposure to the aggressive environments of corrosion and chemical attacks. High temperatures and ultraviolet (UV) rays degrade these coatings in harsh atmospheres and reduce their performance. Optimized epoxy formulations are fabricated with nanoparticles, such as silica and ZnO, to achieve high scratch and abrasion resistance properties and protection from harsh atmospheres and environmental hazards. Silica nanoparticles are used widely in polymer matrices to improve heat resistance, radiation resistance, mechanical, and electrical properties of the polymer materials [5]. Silica nanoparticles effectively improve the hardness, abrasion resistance, and scratch resistance of acrylic-based coatings compared with silica microparticle-based coatings [6]. Nano-silica can provide stiffening strength and toughness to polypropylene materials. In general, a compatible dispersing agent is used to disperse the nano-silica particles in the bulk polymer matrices.

The hydrophobicity, as well as the color stability of uncoated wood samples and epoxy ZnO coated wood samples were evaluated and compared for their color stability. The microstructure morphology and crystal structures results showed that the color stability of the coated wood samples was enhanced by around 50% compared to that of uncoated wood samples. A continuous epoxy ZnO coating with ZnO micro/nanoparticles on wood surfaces was also confirmed during investigation [7]. Ionic liquid-silica/epoxy composites showed higher mechanical and thermal properties compared with epoxy composite containing un-functionalized silica (u-silica). The dynamic mechanical analysis showed that the storage modulus of composites significantly increased with the addition of IL-silica in comparison to that with added u-silica. The incorporation also improved mechanical and thermal properties, of the epoxy composites [8]. The investigation on the effects of nano-SiO_2_ particles on the tensile properties and tensile fracture face morphology of Silica-Filled Phenolic Amine/Epoxy Resin nanocomposites exhibited that the uniform dispersion of SiO_2_ nanoparticles contributed to enhance the tensile performance of nanocomposites. It was also revealed that the addition of 1.5% weight parts of nano-SiO_2_ in epoxy composites enhanced the tensile strength and tensile modulus by 184.1% and 217.2%, respectively [9]. During the study, it was found that incorporation and better dispersion of silica nanoparticles into a polymer matrix could contribute to enhance the thermomechanical properties of the composites and increase their glass transition temperature and thermal conductivity. Silane coupling agent also played an important role to achieve better thermomechanical properties for the composite as compared with that of pure epoxy coating [10]. Epoxy toughening using elastomers can be further enhanced by using nano-silica to fabricate high performance nano-composite. The synergy as toughness and fatigue performance can be improved significantly [11].

The performance of epoxy coatings with different zinc-rich primers has been tested and evaluated over the last few years for offshore applications. All the coatings performed well with zinc silicate primers. Generally, offshore industries use zinc-rich epoxy coatings to inhibit the corrosion of steel pipes from the initial coating history. However, recently developed epoxy-mastic coatings exhibited better performance when applied directly to blast-cleaned steel without a zinc primer [12].

The zinc-rich primer protects the steel surface through cathodic protection, which is illustrated by the electrochemical properties of zinc-rich primers. As these zinc particles corrode, the contact of the coating materials with the substrate (steel) will gradually be lost. After a certain period, the potential of the steel exceeds the protection level. However, the coating may still provide corrosion protection by maintaining the pH under the coating and sealing the pores in the primer to improve the barrier properties [13,14,15]. Silicone incorporation into epoxy resin enhanced the impact strength, thermal resistance, moisture resistance, chemical resistance, and corrosion resistance because of its inherent flexibility, constant stress, partial ionic nature, and excellent dielectric properties [16]. Recently, the adhesion, scratch, and impact of epoxy coatings have been improved considerably by the addition of nanoparticles, such as nano-silica and micron-sized silica pigments. Various epoxy coatings have been developed by incorporating SiO_2_, Zn, and Fe_2_O_3_ into the epoxy matrix system. This experiment suggested the contribution of nanoparticles in significantly improving the corrosion resistance of the applied coating, with Fe_2_O_3_ nanoparticles being the best. SiO_2_ enhances the anti-corrosive properties and mechanical properties. Electrochemical impedance spectroscopy (EIS) measurements indicated that the incorporation of nanoparticles increased the coating resistance significantly.

The epoxy modified with SiO_2_ nanoparticles exhibited a significant enhancement in Young’s modulus [17]. Hongwei et al. [18] dispersed and prepared an epoxy nano-paste by incorporating TiO_2_ particles to investigate the effect of epoxy resin on aluminum alloy. The effect of nano-silica with glycidoxypropyl triemethoxy silane modification or without modification on the epoxy resin has also been studied. The results revealed enhanced hardness in the developed coating because of the incorporation of 1% nano-TiO_2_ and nano-SiO_2_. EIS and the salt spray test showed that the addition of nano-TiO_2_ and nano-SiO_2_ enhanced the corrosion and chemical resistance of the developed coating significantly. The mechanisms for improving the mechanical performance and corrosion resistance were attributed to the coupling agent, which facilitated the incorporation of nanoparticles into the epoxy matrix system [17]. Silane coupling agents to treat the nanoparticle surface before addition to the epoxy matrix also enhanced the dispersion of nanoparticles significantly [19,20,21,22]. This study focused on preparing epoxy nanocomposites using a direct incorporation methodology of nanoparticles into the epoxy matrix of the thermoset resin. A transparent and low viscosity epoxy coating was reinforced with nano-silica and zinc oxide nanoparticles. The mechanical and thermal properties of the produced nanocomposite were reported.

The incorporation and addition of nano-ZnO and nano-SiO_2_ on the properties of the PVA/xylan composite showed enhanced mechanical strength, moisture and oxygen barrier properties, and surface hydrophobic properties. Fourier transform infrared spectroscopy and X-ray diffraction (XRD) revealed the interaction of hydrogen bonds between the nanoparticles, PVA, and xylan, with a concentration of nano-ZnO and nano-SiO_2_ in the composite films of 3%. The tensile strength was significantly to 20.4 and 22.5 MPa, respectively [23]. The author described the effects of synergism in addition to SiO_2_ nanoparticles, such as clay, alumina, titanium, or zirconia, as fillers in epoxy resins. This produced a synergic effect because combining two nanomaterials have greater strength to the adhesive capacity than what can be attained by a single nanomaterial [15].

This study examined the mechanical properties of the epoxy-based coating containing SiO_2_ and ZnO (SNZ1–SNZ3), considering enhancement and performance because of the synergism of two types of nanoparticles, which were reported for different nano-fillers in other applications.

## 2. Materials and Methods

Diglycidyl ether Bisphenol A (DGEBA) type epoxy resin was purchased from Hexion chemicals, Iserlohn, Germany. The curing agent, Aradur D-450, was purchased from Huntsman Advance Materials, Deutschland, Germany. Solvents such as acetone, xylene, and MIBK were purchased locally (Idael Chemicals, Riyadh, KSA). Silica nanoparticles (10–20 nm particle size) and ZnO nanoparticles (<50 nm particle size) were purchased from Sigma-Aldrich (St. Louis, MO, USA) with catalog numbers 637,238 and 677,450, respectively. All the materials were used as received without further purification. 

Coating formulations with a fixed percentage of silica nanoparticles and variable percentages of ZnO nanoparticles were prepared using stoichiometric balanced amounts of epoxy resin and hardener. The procedure of the formulation was followed similarly and is described in our previous published article [24]. Table 1 lists the complete formulation ingredients of the formulation and their quantities.

Fourier-transform infrared spectroscopy (FT-IR) measurements were performed on samples to observe any chemical changes in coatings before and after exposure to aggressive environment.

X-ray diffraction (XRD) Bruker D8 discover (Cu Kα, Bruker, Billerica, MA, USA) was utilized to verify the nanoparticles in prepared coating system. All the XRD scans were in 2 θ range of 10–80° degrees at a scan speed of 2°/min at room temperature. The XRD was used at 40 kV and 40 mA as operating conditions. Q600 by TA instruments (New Castle, DE, USA) was used to examine the temperature profile and decomposition temperatures. The samples were heated from room temperature (25 °C) to 600 °C at a 10 °C/min ramping rate under an N_2_ environment.

Conventional testing, including as a Koenig pendulum tester (model 707/K, ASTM D-4366), a scratch tester (model 705, ASTM D-7027, Sheen Instruments, Surrey, UK), and a Gardener impact tester (model IG-1120, ASTM D-2794, BYK, Columbia, SC, USA), was employed to determine the mechanical properties of the coatings. A Koenig pendulum defines the surface hardness by counting the number of oscillations of the surface. A scratch tester was used to measure the coating resistance. The impact resistance of the coatings was determined by dropping a weight from various heights, making an indent on the coating surface until coating rupture. The results of these tests are shown as the average results of three test specimens.

The nanomechanical properties of coatings were determined using a Nanotest platform from Micromaterials, Wrexham, UK. A Berkovich type indenter was used to extract the nanomechanical properties of the coatings, such as the elastic modulus and hardness. The properties were measured by subjecting the coatings to a maximum load of 250 mN using a load control program. An increase of 1 mN/s was applied until the maximum load was achieved. The maximum load of 250 mN was held constant for 60 s to check the creep behavior of the coatings under a maximum load, followed by unloading at the same rate of 1 mN/s until the complete load was removed. At least 10 indentations were taken on each sample to obtain consistency in the results, and the presented results are the averaged final results.

The abrasion resistance of the coatings was determined using a dual rotary platform Taber Abrader (model 5155, Sheen Instruments, Surrey, UK). The coated panels were subjected to abrasion using CS-17 wheels for 500 cycles at 60 cycles per minute. After completing 500 cycles, CS-17 wheels were refaced (50 cycles). After refacing, another 500 cycles at the same speed were performed on the samples. With the completion of 1000 cycles on each sample, the differences in the initial and final readings were recorded. At least three tests were carried out on each sample, and the results are averaged.

## 3. Results and Discussion

### 3.1. FTIR Analysis

**Figure 1:**
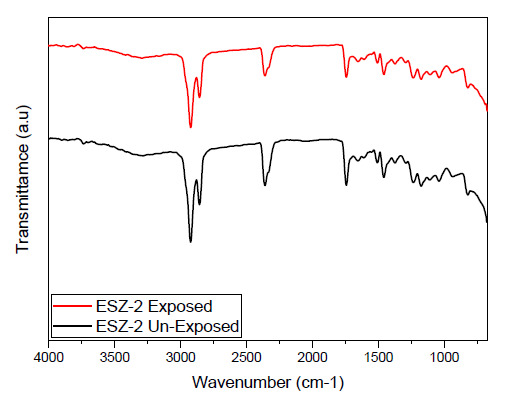
FTIR spectrum of ESZ-2 coatings unexposed and after 30 days’ exposure to saline environment.

FTIR characteristic peaks were obtained on ZnO incorporated epoxy coating samples. Figure 1 shows the obtained FTIR graphs for ESZ-2 coating sample and the same sample after exposure to 3.5% NaCl solution for 30 days. The corresponding peaks obtained for the unexposed samples are described according to the wavenumbers. The broad band in the range of (3100–3600 cm^−1^) is related to stretching vibration of OH. The peaks corresponding to the range (2800–2990 cm^−1^) are related to C/H vibration for epoxy. The peaks at 1580–1650 cm^−1^ correspond to the primary amine (NH), the peak at 1509 cm^−1^ corresponds to the N-O stretching indicating nitro compounds, peaks corresponding to 1458 cm^−1^ and 1230 cm^−1^ shows C-C and C-O stretching, 1000–1100 cm^−1^ correspond to C-N group and 830 cm^−1^ is related to the 1,4-substitution of aromatic ring of the DGEBA resin.

The FTIR results obtained for coatings after 30 days’ exposure to the saline solution suggest no changes in the functional group and no new peak appeared. In the exposed environment the aggressive ions can interact with –OH and –NH groups and cause the degradation of the coating. The absorption of water in the coatings also causes the degradation which could result an increase in intensity of the bands associated with the hydroxyl group. Another important cause affecting the coating degradation is associated to ultraviolet radiations; these radiations cause the primary bonds in the polymeric chains to break thus generating free radicals. This process is called photolysis. These free radicals then react with oxygen and other aggressive species, causing a chain scission reaction [25]. In epoxy chain the methyl group is most prone to ultraviolet attack which leads to its conversion to carbonyl [26]. This photolysis reaction is very limited in the case of coatings with ZnO. Our results up to 30 days of exposure suggest no degradation in the coatings, as no change in intensity of the FTIR peaks was observed after the exposure period. This stability of the coating is because of ZnO which absorbs large amount of UV radiation thus preventing chain scission [27]. Muhammad et al. [28] reported epoxy coatings prepared with different types of fillers, i.e., ZnO, Fe_2_O_3_, TiO_2_, and graphite. They reported hydrolytic degradation in all the coatings except the coatings with ZnO. The coating with ZnO was the most stable and only showed signs of degradation after 90 days of exposure.

### 3.2. X-ray Diffraction (XRD)

**Figure 2:**
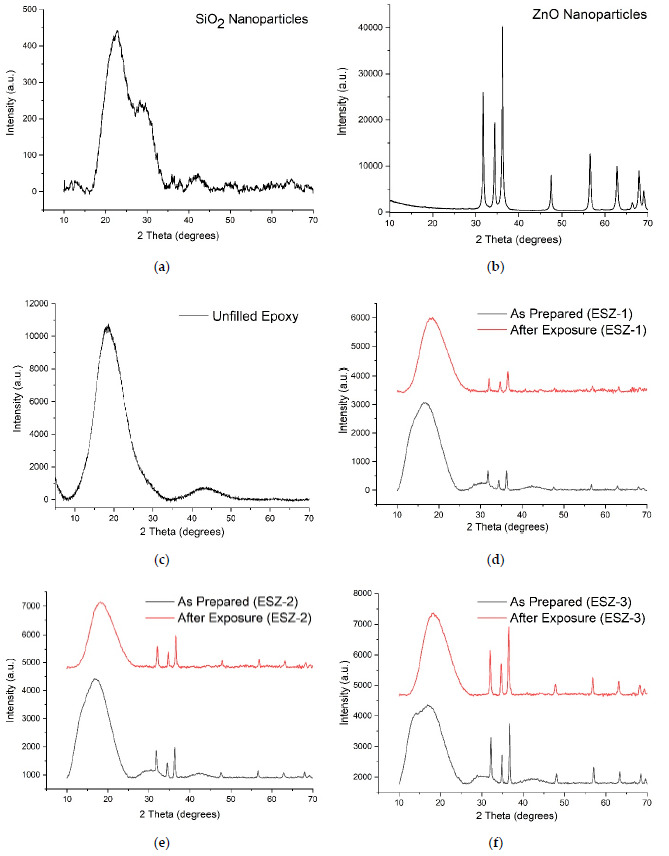
XRD patterns (**a**,**b**) SiO_2_ and ZnO nanoparticles (**c**) unfilled epoxy, (**d**–**f**) composite coatings ESZ-1–ESZ-3.

Figure 2 shows the XRD patterns of the nanoparticles and the nanocomposite films with 5 wt.% SiO_2_ and varying concentrations of ZnO nanoparticles. The XRD peaks belong to the hexagonal ZnO (wurtzite) crystal structure (JCPDS # [01–089–1397]. The peak positions and their corresponding planes were 31.73°/ZnO (1 0 0), 34.40°/ZnO (0 0 2), 36.22°/ZnO (1 0 1), 47.49°/ZnO (1 0 2), and 56.56°/ZnO (1 1 0). The peak intensity of ZnO increased with increasing ZnO nanoparticle content in the epoxy polymer. The XRD pattern of SiO_2_ showed no sharp peaks, which confirms the amorphous structure of the silica nanoparticles. The incorporation of ZnO to the epoxy polymer matrix increases the crystallinity of the resulting composite coatings which increased with increase in the ZnO loading.

The average d-spacing for SiO_2_ increased after incorporation in the epoxy matrix [24]. The change in d-spacing is an essential parameter for determining the dispersion mechanism that occurs by either by intercalation or exfoliation. The exfoliation mechanism of the dispersion should occur when the d-spacing is higher than 10 nm [29]. The d-spacing for 5 wt.% nanosilica incorporated in the epoxy matrix increased from 4.52 to 5.31 Å, confirming that dispersion occurred via the intercalation mechanism, as reported by Gurusideswar et al. [30]. However, no substantial changes in the d-spacing of the ZnO nanoparticles were observed after dispersion in the epoxy coating matrix at various concentrations. The crystallite sizes were calculated using Scherer’s equation. Table 2, Table 3 and Table 4 report the various crystallite sizes of the nanoparticles, the as-prepared composite coatings, and the composite coating after exposure to the saline solutions, respectively. The average crystallite size of the exposed samples decreased for the SNZ-1 and SNZ-3 samples whereas it increased for the SNZ-2 samples.

### 3.3. Thermogravimetric Analysis (TGA)

**Figure 3:**
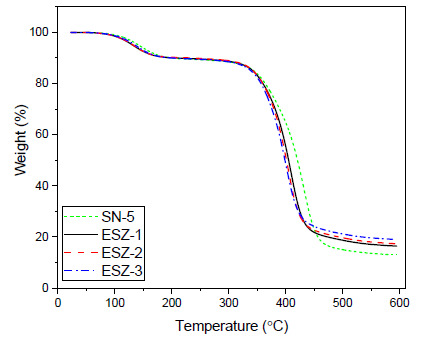
Thermogravimetric analysis decomposition profile of SN-5 (5% SiO_2_ nanoparticles), ESZ-1 (5% SiO_2_ nanoparticles, 1% ZnO), ESZ-2 (5% SiO_2_ nanoparticles, 2% ZnO), and ESZ-3 (5% SiO_2_ nanoparticles, 3% ZnO).

Figure 3 shows the TGA curves of all the prepared coatings, and Table 5 lists the decomposition temperatures at different weight percentages. A similar weight loss pattern was observed in all the samples, with initial decomposition starting from approximately 100 °C until 250 °C. The initial weight loss typically occurs because of the removal of volatiles trapped in the epoxy crosslinked structure or decomposition of the low molecular weight resin fractions. This can also occur because of the residual unreacted two-part components.

The major decomposition of the nanoparticle-modified coatings started from 300 °C and extended to 500 °C. In this decomposition profile, with the addition of ZnO, the decomposition temperatures were reduced compared with the coating without ZnO nanoparticles. This second decomposition wave above 300 °C results from the decomposition of the main resin chains, the degradation temperature for each sample at 15, 25, 50, and 75% weight loss were recorded, as summarized in Table 5. Coatings without ZnO nanoparticles withstood the highest temperature at all weight loss percentages because of the tendency of silica to act as a thermal insulator [31,32]. Arabli et al. [33] also reported in their finding that incorporation of silica into epoxy improved the thermal stability. With the incorporation of silica nanoparticles, the initial decomposition temperature and maximum degradation temperature both increased. With the addition of ZnO nanoparticles, starting with 1%, the degradation profile showed lower resistance to thermal insulation, and coatings started to decompose at lower temperatures. With increasing ZnO percentage, the thermal properties of the coatings weakened further, as shown in the Table 5. The temperature of 50% and 75% weight loss of the coatings without ZnO was 420 °C and 477 °C, respectively. In contrast, it was reduced significantly to 398 °C and 438 °C with 3% ZnO particles, which correspond to a 5% and 8% decrease, respectively, compared with the coating without ZnO nanoparticles. Table 5 lists the percentage residue after complete decomposition of samples at 600 °C. The lower degradation temperature of the coatings was attributed to the ZnO nanoparticles, which alters the final properties of the coatings by imparting a catalytic effect on the thermal resistance, which can be observed, even with very low fractions of nanoparticles.

These results are in agreement with those reported in literature [34] and our previous findings [35] where addition of ZnO nanoparticles decreased the coatings thermal properties by lowering the decomposition temperatures at different weight percentages because ZnO has the tendency to form free oxygen and oxygen vacancies. These oxygen vacancies are capable of absorbing electrons resulting in catalytic positions [36]. Hsu et al. [37] reported that with the addition of ZnO the decomposition temperatures were decreased; he also reported that with the increase in ZnO percentage the thermal properties of epoxy were further reduced. Also reported by Baghdadi et al. [38], the addition of 2.5 wt.% ZnO into epoxy matrix decreased the onset temperature of epoxy. This happens because of the tendency of ZnO to accelerate the degradation by acting as a thermal transport medium.

### 3.4. Mechanical Properties and Nanoindentation

The coatings after complete curing were subjected to mechanical characterization, including the pendulum hardness, scratch, and impact resistance. With the addition of 2% ZnO nanoparticles, the mechanical properties increased slightly. The pendulum hardness and impact resistance were 147 oscillations and 136 lb/in^2^, respectively, compared with 118 oscillations and 128 lb/in^2^ with 0% ZnO. The scratch resistance up to this percentage was relatively unchanged at 9 kg. With the addition of 3% ZnO, the coating properties deteriorated, as shown in the Table 6. This was attributed to the high loading of nanoparticles in the matrix resin. At lower percentages, the dispersion of nanoparticles was easy. The filler–filler interactions were more favorable at higher loadings than the filler–polymer interaction, which leads to poor mechanical resistance [39].

**Figure 4:**
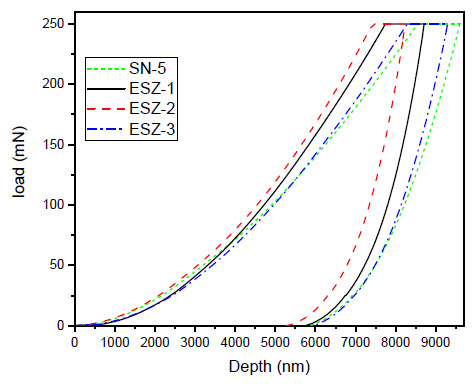
Load vs. depth curves.

Nanomechanical characterization was performed on all the prepared coating samples to extract coating hardness and modulus by employing nanoindentation technique using Berkovich type indenter. For the purpose of evaluating the coating properties, all the prepared coating samples were subjected to a maximum load of 250 mN using load control program. As the indenter penetrates into the material surface, the underneath material deforms and counter the applied load by the indenter. Therefore, the typical loading and unloading curves obtained during indentations, Figure 4 shows the load vs. depth curves. The loading graphs showed that loading was achieved quite smoothly without any discontinuities, suggesting no cracking on the coating surface during the loading process. All the graphs with the addition of ZnO nanoparticles shifted to lower depth values except the SN-5 coating, which did not contain ZnO nanoparticles, suggesting an increase in load-bearing capacity of the coatings.

The maximum depths during the experimental procedure for the SN-5, ESZ-1, ESZ-2, and ESZ-3 samples were 9594, 8707, 8230, and 9292 nm, respectively. It can be seen from Figure 4 that incorporation of ZnO nanoparticles showed positive effect in increasing nanomechanical properties of the coatings. The coating containing 2% ZnO showed the lowest depth penetration. Further increases in ZnO percentage reversed the effects of nanoparticle addition, where the maximum depth penetration by the indenter was higher for 3% ZnO nanoparticle addition followed by SN-5 with 0% ZnO nanoparticles.

The load vs. depth curves were analyzed using the inbuilt software provided by ‘Micro Materials’ to extract the hardness and elastic moduli. These values were derived using the following equations, as reported elsewhere [40]:

(1) $$H=\frac{F_{max}}{A}$$

(2) $$E_{r}=\frac{1-\upsilon^{2}}{E}+\frac{1-\upsilon_{i}^{2}}{E}$$

where H is the hardness, *F_max_* is the maximum load, A is the projected area at maximum load, E is the sample modulus, *ν* is the Poisson ratio (0.35 for polymer), *E_r_* is the reduced modulus (obtained from the machine), Ei is the diamond indenter modulus (1141 GPa), and *ν_i_* is the Poisson ratio of the indenter (0.07).

**Figure 5:**
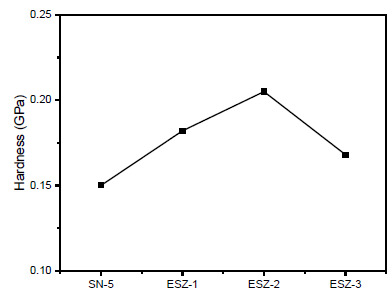
Indentation hardness of the coatings.

**Figure 6:**
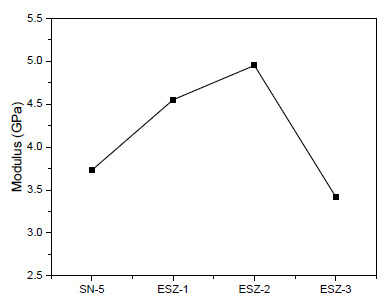
Indentation modulus of the coatings.

In order to compare the obtained properties after nanomechanical testing, the hardness and modulus of all the coating samples tested are shown in Figure 5 and Figure 6, respectively. With the addition of ZnO nanoparticles, the hardness and modulus of both coatings increased. The addition of 3% ZnO retarded the properties but the obtained values are still higher than coatings without ZnO. With SN-5 (5% silica and 0% ZnO), the hardness and modulus were 0.15 and 3.73 GPa, respectively. The highest values of hardness and modulus in all the prepared coatings were obtained for ESZ-2 (5% silica and 2% ZnO), which were 0.205 and 4.95 GPa, respectively.

The hardness and modulus increased because of nanoparticle addition, mainly because of free volume reduction in the epoxy matrix after crosslinking. The added nanoparticles take up the free volume that enhances the material properties [41,42,43]. The results were in agreement with those obtained by conventional mechanical testing, where the coating properties, such as the pendulum hardness, scratch, and impact, were higher for the coating with 2% ZnO. The further increase in nanoparticle loading resulted in inferior mechanical properties.

The dispersion of nanoparticles is a challenging task because of the higher filler to filler attraction; these nanoparticles are prone to agglomeration when used in higher quantities. The deterioration of the mechanical properties of the coatings using 3% ZnO nanoparticles was attributed to this phenomenon. The percentage beyond 2 wt.% ZnO reflects the negative effect in terms of the material properties because of failure to achieve better dispersion of nanoparticles as they become more prone to agglomeration [44]. Thipperudrappa et al. [45] reported in their findings that optimum loading ratio at which they achieved maximum properties was 2%, beyond this percentage the material properties decreased. Li et al. [46] also reported the similar findings where decrease in properties was witnessed after exceeding 2% loading ratio.

**Figure 7:**
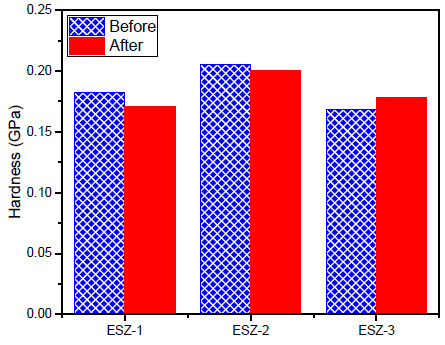
Indentation hardness before and after exposure.

**Figure 8:**
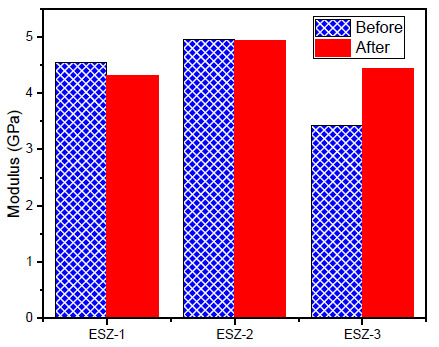
Modulus before and after exposure.

The coating properties were re-examined after exposing them to a marine environment. The samples were exposed to a 3.5% NaCl solution for 30 days. After 30 days, the samples were dried and analyzed to examine the effects of NaCl exposure on the hardness and modulus. The graphical representation for the results of both before and after exposure, for hardness and modulus of coatings containing ZnO, is shown in Figure 7 and Figure 8.

After exposure, the coating properties with 1% ZnO decreased, whereas with 2% addition, the properties remained similar without significant reduction. For 2% ZnO addition, the hardness and modulus were 0.205 and 4.95 GPa, respectively. After exposure, the values obtained were 0.200 and 4.93 GPa, respectively, which can be considered the same. A noticeable change was recorded with 3% ZnO addition after exposure to the 3.5% NaCl solution, where the hardness increased from 0.16 to 0.17 GPa. Similarly, the modulus increased from 3.42 to 4.43 GPa. Similar results were obtained by Cristea et al. [47] where the indentation modulus and hardness were increased after artificial weathering because of the increased stiffness in the coatings. They concluded that artificial exposure affects the plasticization phenomenon by moisture take-up in the coatings. Furthermore, the filler distribution in the polymer matrix can affect the indentation results significantly. The polymer material subjected to accelerated weathering showed an increase in the modulus [48].

### 3.5. Creep Resistance

**Figure 9:**
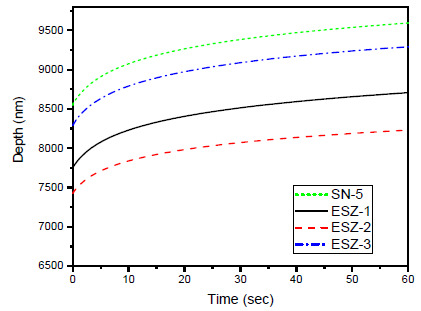
Creep representation of a 60 s holding period.

**Figure 10:**
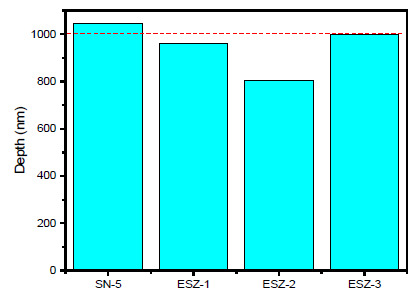
Depth penetration during the 60 s holding creep period.

At a maximum load of 250 mN, the load was held for 60 s, which subjects the samples to creep behavior to check the effect of nanoparticle inclusion on time-dependent deformation. Generally, creep occurs in two stages in nanoindentation, i.e., transient and steady. The initial stage is transient. Most creep deformation occurs in this stage, followed by a steady stage that is relatively stable. Figure 9 presents the creep curve for all the samples. The addition of ZnO nanoparticles alters the creep properties significantly in a similar manner to the depth penetration observed in the load vs. depth curves. Figure 10 shows the depth penetration of the indenter in the overall holding period, where the minimum depth penetration was observed for ESZ-2 compared with all other samples, including samples without ZnO.

Creep deformation in a polymer depends on the applied load and the duration of that applied load (time dependent) [49]. The increase in creep resistance with the addition of ZnO was attributed to its polycrystalline nature. A similar effect is reported elsewhere [35], where an increase in creep resistance was witnessed because of ZnO addition. This usually occurs because of the lower chain flexibility after incorporating nanoparticles in the matrix resin, which causes a decrease in the creep deformation. Another reason for increase in creep could be attributed to strong interfacial adhesion between the nanoparticles and epoxy resin matrix [50] which can be achieved when the dispersion of nanoparticles is sufficient. This strong interaction between resin and nanoparticles results in an increased crosslinked density [51]. Also, the addition of nanoparticles acts as a barrier against an external indentation force [41], resulting in superior properties because of the excellent load transferability between the matrix and filler.

### 3.6. Abrasion Resistance

The abrasion resistance of the coatings according to ASTM D4060 after adding silica and ZnO nanoparticles was evaluated using a Taber abrader running for 1000 cycles on each coating with refacing of the wheels at 500 cycles for 50 cycles. The following results were obtained using the following equation:

(3) $$Mass\;Loss=M_{initial}-M_{damaged}$$

where *M_initial_* is the intact coating mass and *M_damaged_* is the coating mass after abrasion.

**Figure 11:**
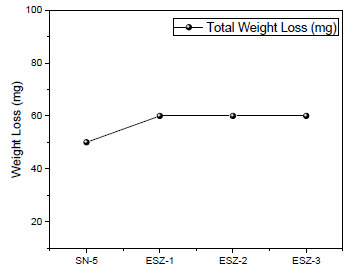
Mass loss profile for the nanoparticle-modified coatings.

With the addition of ZnO (Figure 11), a slight increase in abrasion resistance was recorded after 1000 cycles. The percentage of ZnO did not induce any changes in abrasion resistance because the results obtained with 1%, 2%, and 3% addition of ZnO were the same. Hence, the percentage increase in ZnO did not produce any changes in the final abrasion resistance of the coatings.

## 4. Conclusions

The effects of SiO_2_ and ZnO synergism on the mechanical and thermal properties of epoxy coatings were studied. The samples were prepared by dispersing nanoparticles in epoxy resin with sonication. Varying the percentage of ZnO with a fixed percentage of SiO_2_ was used. The XRD results showed that the crystallinity of the composite coatings increased with increase in the ZnO content. The results showed that the addition of ZnO nanoparticles along with the silica nanoparticles increased the final mechanical properties with up to 2% ZnO addition, and nanoindentation indicated an increase in coating hardness. Nanoindentation results revealed a 33% increase in the maximum hardness compared with the coatings without ZnO. The prepared coatings were also subjected to saltwater exposure, where a decrease in properties was minimal for coatings with 2% ZnO. Conventional mechanical testing showed that the coating with 2% ZnO had the highest impact, scratch, and pendulum hardness. The addition of ZnO had a negative influence on the final properties of the coatings. Increasing the amount of ZnO degraded the thermal stability compared to the coating without ZnO. Furthermore, the coatings without ZnO had a higher degradation temperature than the coatings with ZnO.

## Figures and Tables

**Table 1:** Complete formulation ingredients along with their quantity in the stoichiometric balance of the epoxy and hardener.

Formulation Code	Epoxy	MIBK (mL)	Xylene (mL)	Silane (gm)	SiO_2_ (gm)	ZnO (gm)	D-450
Neat	83.34	8	8	2.0	0	0	16.66
ESZ-1	83.34	8	8	2.0	5	1	16.66
ESZ-2	83.34	8	8	2.0	5	2	16.66
ESZ-3	83.34	8	8	2.0	5	3	16.66

**Table 2:** Crystallite size of the nanoparticles using Scherer’s formula.

Sample	Peak Position (2θ)	FWHM	d-Spacing (Å)	Crystallite Size (nm)	Average Crystallite Size (nm)
SiO_2_	22.41	5.67	3.96	1.41	1.94
	29.14	5.4	3.06	1.5	
	41.93	2.88	2.15	2.92	
ZnO	31.73	0.28	2.82	28.89	27.07
	34.4	0.29	2.6	28.61	
	36.22	0.3	2.48	27.96	
	47.49	0.33	1.91	26.04	
	56.56	0.37	1.63	23.82	

**Table 3:** Crystallite size of the nanoparticles in the composites (unexposed) using Scherer’s formula.

Sample	Peak Position (2θ)	FWHM	d-Spacing (Å)	Crystallite Size (nm)	Average Crystallite Size (nm)
SNZ-1	31.77	0.36	2.81	22.40	26.26
	34.42	0.31	2.60	26.15	
	36.28	0.32	2.47	25.80	
	47.61	0.33	1.91	26.37	
	56.65	0.29	1.62	30.57	
SNZ-2	31.79	0.40	2.81	20.44	22.97
	34.44	0.35	2.60	23.36	
	36.28	0.35	2.47	23.38	
	47.55	0.39	1.91	21.95	
	56.63	0.35	1.62	25.71	
SNZ-3	32.26	0.32	2.77	25.92	26.87
	34.91	0.30	2.57	27.90	
	36.75	0.30	2.44	27.47	
	48.06	0.35	1.89	24.50	
	57.12	0.31	1.61	28.55	

**Table 4:** Crystallite size of the nanoparticles in the composites (exposed) using Scherer’s formula.

Sample	Peak Position (2θ)	FWHM	d-Spacing (Å)	Crystallite Size (nm)	Average Crystallite Size (nm)
SNZ-1	32.06	0.37	2.79	22.22	23.62
	34.75	0.33	2.58	25.22	
	36.56	0.38	2.45	21.94	
	47.82	0.32	1.90	26.85	
	56.93	0.41	1.62	21.863	
SNZ-2	32.06	0.35	2.79	23.36	24.53
	34.73	0.35	2.58	23.80	
	36.56	0.36	2.45	23.12	
	47.86	0.31	1.90	27.66	
	56.91	0.36	1.62	24.73	
SNZ-3	32.06	0.35	2.79	23.20	22.73
	34.69	0.34	2.58	24.06	
	36.54	0.36	2.46	22.82	
	47.84	0.43	1.90	19.84	
	56.89	0.38	1.62	23.74	

**Table 5:** Degradation temperatures at different weight loss percentages.

Formulation	Degradation Temperature 15% Loss (°C)	Degradation Temperature 25% Loss (°C)	Degradation Temperature 50% Loss (°C)	Degradation Temperature 75% Loss (°C)	Residue (%)
SN-5	344.10	377.80	420.90	477.03	12.97
ESZ-1	342.16	372.32	405.23	434.79	16.43
ESZ-2	343–01	370.82	401.45	434.55	17.33
ESZ-3	339.25	367.46	398.83	438.43	19.07

**Table 6:** Mechanical properties of the nanoparticle-modified coatings.

Formulation Code	Thickness (µm)	Pendulum Hardness (Oscillations)	Scratch Resistance (kg)	Impact Strength (lb/in^2^)
SN-5	65 ± 5	118	9	128
ESZ-1	60 ± 5	136	8	120
ESZ-2	65 ± 5	147	9	136
ESZ-3	65 ± 5	135	6	96

## Data Availability

The data presented in this study are available on request from the corresponding author.
